# Attentive-executive functioning and compensatory strategies in adult ADHD: A retrospective case series study

**DOI:** 10.3389/fpsyg.2022.1015102

**Published:** 2022-10-13

**Authors:** Martino Ceroni, Stefania Rossi, Giorgia Zerboni, Elena Biglia, Emiliano Soldini, Alessia Izzo, Lucia Morellini, Leonardo Sacco

**Affiliations:** ^1^Neuropsychological and Speech Therapy Unit, Neurocenter of Southern Switzerland, EOC, Lugano, Switzerland; ^2^Competence Centre for Healthcare Practices and Policies, Department of Business Economics, Health, and Social Care (DEASS), University of Applied Sciences and Arts of Southern Switzerland, Manno, Switzerland; ^3^Faculty of Biomedical Sciences, Università della Svizzera italiana, Lugano, Switzerland

**Keywords:** adult ADHD, cognition, executive functions, attention, socio-demographic factors

## Abstract

**Background:**

Adults with ADHD exhibit a neuropsychological profile that may present deficits in many cognitive domains, particularly attention and executive functions (EFs). However, some authors do not consider executive disfunction as an important part of the clinical profile of the syndrome; this could be related to the use of inappropriate neuropsychological tests, probably not adapted and not sufficiently ecological. Moreover, new data are required on specific correlation of attentive-executive symptoms with socio-demographic factors. Therefore, the aim of this study is to analyze the neuropsychological performance of a group of adults with ADHD, also evaluating the influence of gender, age and education level.

**Methods:**

We retrospectively collected health-related personal data of 40 adult ADHD patients, clinically diagnosed and evaluated *via* a battery of 4 neuropsychological tests and 1 self-administered questionnaire. Gender, age and years of education differences were assessed.

**Results:**

Attention and EFs deficits have been highlighted mainly on the d2-R and 5-point neuropsychological tests, which therefore seem to be more sensitive in measuring the attention-executive dysfunction in an adult ADHD population, than TAP Go/No-go and ROCFT. ADHD patients also manifested subjective behavioral impulsivity disorders on BIS-11. There were no statistically significant gender differences in cognitive performance. On the contrary, younger patients performed worse on subscales TAP Go/No-go errors and 5-points number of drawings, while participants with a higher education level performed better on subscales d2-R speed of execution and d2-R errors. This supports a reduction in the number of errors and the execution time as a function of older age and a higher level of education. Finally, patients with higher education also self-reported greater impulsivity in planning.

**Conclusion:**

Our preliminary findings suggest that adult ADHD is not a lifelong stable disorder, but it may change over time. Moreover, attention-executive deficits may be influenced and partially counterbalanced by experience (i.e., advancing age) and a higher level of education. This could underlie the development of specific psycho-behavioral and cognitive compensatory strategies. The use of self-administered questionnaires is therefore recommended to highlight attentional and executive difficulties that may not result in neuropsychological tests.

## Introduction

According to the fifth edition of the diagnostic and statistical manual of mental disorders (DSM-5; American Psychiatric Association, 2013), attention deficit hyperactivity disorder (ADHD) is a neurodevelopmental disorder characterized by inattention, impulsivity, and hyperactivity. These components can all be present, thus defining ADHD combined type (ADHD-C), or be singularly predominant in ADHD hyperactive–impulsive type (ADHD-HI) or inattentive type (ADHD-I). These ADHD subtypes certainly have a clinical value to describe the functioning and the type of behavior, but they do not categorize subgroups with adequate long-term stability to motivate a subdivision of the disorder into distinct forms (Willcutt et al., 2012).

Contrary to what was claimed in previous years, this disorder is not limited to children and adolescents, but for approximately 50% of patients ADHD-related problems persist during adulthood to varying degrees (Barkley, 2009; Asherson et al., 2014). Based on DSM-5, over 5% of children and 2,5% of adults suffer from ADHD disorder (male-to-female ratio of 2:1 in children and 1.6:1 in adults), which is associated with a high risk of developing personality disorders and serious impairments in academic, health, occupational and social domains (American Psychiatric Association, 2013). Additionally, persistence of symptoms in adulthood is more common in females (Biederman et al., 2010; Hinshaw et al., 2012), who are more likely to present attention deficits, while males are more inclined to present hyperactivity and impulsivity (Willcutt et al., 2012). The clinical manifestations of ADHD disorder present individual differences and, evolve from childhood to adulthood. Indeed, hyperactivity and impulsivity are considered as “first symptoms” – these traits are particularly evident in childhood - while attention deficit prevails with advancing age (Biederman et al., 2000). Other symptoms, including restlessness, disorganization, problems in emotion regulation and other cognitive difficulties are also prominent in adults (Alderson et al., 2013; Karam et al., 2015; Mowinckel et al., 2015). According to the systematic review made by Onandia-Hinchado and collaborators (2021), adults ADHD exhibit a neuropsychological profile that may present deficits in attention, processing speed, executive functions (EFs), arithmetic abilities, verbal memory, reading skills and social cognition. Furthermore, the cognitive performance of ADHD patients is characterized by a high inter-individual variability. Specifically, both adults and children with ADHD show attentional variability in reaction time (Tamm et al., 2012; Sjöwall et al., 2013), while it is mainly adults with ADHD who exhibit variability in executive functioning (Gonzalez-Gadea et al., 2013). Consequently, attention and EFs, meant as a set of cognitive processes which are necessary for conscious and goal-oriented problem solving (such as monitoring/updating in working memory, inhibitory control, mental flexibility and planning; Diamond, 2013) are the most studied cognitive domains in adult ADHD patients. Attention seems to be compromised in all the modalities, such as alertness, vigilance and even sustained, selective and divided attention (Tucha et al., 2017). In addition, adults with ADHD show significant deficits in many of specific EFs processes (Barkley, 1997; Biederman et al., 2006; Brown, 2006; Barkley and Murphy, 2010, 2011; Adler et al., 2017), which are also confirmed by neuroimaging studies that support the relationship between adult ADHD and executive dysfunction (Cortese et al., 2012; van Ewijk et al., 2015; Abramov et al., 2019). Moreover, Silverstein et al. (2020) concluded that symptoms of ADHD were strongly correlated and predictive of general EFs troubles.

However, some authors do not consider executive dysfunction as an important part of the clinical profile of ADHD syndrome (Nigg et al., 2005; Willcutt et al., 2005; Marchetta et al., 2008). Part of this non-homogeneous results between studies could be interpreted by an inappropriate use of neuropsychological tests, often not enough adapted to the study population and not sufficiently ecological (i.e., not exactly equivalent to a particular situation in everyday life, requiring specific cognitive skills; Barkley and Murphy, 2011).

As mentioned above, it could be very challenging to make a correct diagnosis of ADHD by identifying all the behavioral and cognitive facets that describe the specific profile of the patient. Indeed, ADHD is often not detected in time or, even worse, misdiagnosed (Giuliano and Geyer, 2017). Moreover, clinicians must accurately differentiate ADHD from other clinical conditions that are often comorbid (Faraone et al., 2006), such as bipolar disorder, borderline personality disorder, substance abuse disorder, anxio-depressive disorder, etc. (Ohnishi et al., 2019). Therefore, the diagnosis of adult ADHD must be made using multiple sources of information, including self- and hetero-anamnesis, clinical interviews, childhood documentation (i.e., school reports, judgments, etc.), questionnaires and neuropsychological tests that include an exhaustive evaluation of the socio-cognitive functioning, in order to examine the clinical profile in detail (Gibbins and Weiss, 2007; Paris et al., 2015; Sibley, 2021). Accordingly, in our service we have devised a specific clinical procedure for the diagnosis of ADHD in adults, which includes a battery of neuropsychological tests and self-administered questionnaires. In the present study, we therefore proceed to analyze part of these data, collected from a group of adult patients formally diagnosed with ADHD in our unit, with the aim of defining specific patterns of functioning and also of identifying which tests are more sensitive to cognitive characteristics of the disorder. Additionally, since to our knowledge there are few studies that have directly investigated how the cognitive performance of ADHD adults on specific neuropsychological tests/questionnaires varies as a function of socio-demographic factors, we want to assess the relations between socio-cognitive functioning and three key socio-demographic factors: gender, age and level of education. Indeed, there is little evidence in the existing literature comparing cognition in males and females affected by ADHD and most of these are on children or adolescent (Carucci et al., 2022). Other few studies have correlated the clinical manifestation and/or onset of the disorder with age, gender, socio-economic status, school grade, occupation, first-born status, parents’ age, etc. (Bernfort et al., 2008; Canals et al., 2018; Salvi et al., 2019). In conclusion, our results will complement the existing literature in characterizing the cognitive functioning of adult ADHD and could also provide new and interesting information on the correlations of cognitive symptoms with socio-demographic factors.

## Materials and methods

### Patients’ data inclusion

The health-related personal data of adult patients (≥ 18 years old), formally diagnosed with ADHD in our institution (Neuropsychological and Speech Therapy Unit, Neurocenter of Southern Switzerland) were retrospectively collected and included into this case series study, after reporting by the clinicians who made the diagnosis (between February 2019 and May 2022).

The diagnosis was made by experienced neuropsychologists and neurologists following the latest guidelines and according to the DSM-5 criteria, also including structured diagnostic interviews (i.e., no screening procedures for symptoms only) such as the Barkley adult ADHD rating scale-IV (BAARS-IV; Barkley, 2011a), the Barkley functional impairment scale (BFIS; Barkley, 2011b; i.e., no screening procedures for symptoms only) and/or the diagnostic interview for ADHD in adults 2.0 (DIVA 2.0; Kooij and Francken, 2010).

Exclusion criteria for not including data from an ADHD patient were: psychotic or neurologic condition (e.g., seizure disorder, physical brain injury, bipolar disorder, schizophrenia; not including ADHD with common comorbidities such as internalizing, externalizing, and learning disorders); concurrent major depression at the moment of the neuropsychological evaluation; diagnosis of intellectual disability (IQ < 70); existence of a documented refusal.

Finally, the collected data came from a non-medicated group of patients, whom did not take specific ADHD pharmacotherapies such as Methylphenidate or Lisdexamfetamin at the moment of data collection and diagnosis.

### Evaluation

Data related to neuropsychological assessments performed by ADHD adults have been collected retrospectively. Health-related personal data were collected between February 2019 and May 2022.

*Cognitive status assessment*: The attentive-executive functioning of the patients was explored through a battery of selected and validated tests and questionnaires, which particularly consisted of 4 neuropsychological tests handled by a qualified neuropsychologist and a self-administered questionnaire. The neuropsychological tests used were the d2-R (normative values of Ciancaleoni and Fossati, 2013), the Go/No-go subtest from the Test Battery for Attentional Performance (TAP version 2.3; normative values of Ruggeri, 2012), the 5-points test (normative values of Cattelani et al., 2011) and the Rey-Osterrieth Complex Figure Test (ROCFT; normative values of Caffarra et al., 2002); while the self-administered questionnaire is the Barratt Impulsiveness Scale (BIS-11; normative values of Fossati et al., 2001). Subsequently, attention and processing speed functions were assessed by the d2-R, which gives an appreciation of selective/focused and sustained attention skills and has a good concurrent validity with ADHD (Brickenkamp et al., 2010; Yato et al., 2019). The inhibitory control was instead assessed using the subtest Go/No-go of the TAP (Zimmermann and Fimm, 2002), which was chosen to evaluate the cognitive performance. This choice is in accordance with the recent literature, specifically for the gender differences in cognitive functioning in adult ADHD (Stibbe et al., 2020). Planning, strategy and monitoring/updating were instead evaluated through the 5-point test (Goebel et al., 2009) and the copy of the ROCFT (Rey and Osterrieth, 1941), both widely used in the literature to study executive functioning in ADHD (Seidman et al., 1998; Murphy et al., 2001; Sami et al., 2004; Barkley et al., 2008). Finally, behavioral disinhibition and impulsivity were also recorded by the BIS-11 (Patton et al., 1995), which is commonly administered to evaluate the actual subjective functioning of ADHD patients in everyday life (Nandagopal et al., 2011; Speranza et al., 2011; Mersin Kilic et al., 2020).

*Socio-demographic variables*: age, gender and level of education (number of years of education) were collected.

### Statistical analysis

Since there was no specific control group in this retrospective case series study, we convert the raw scores obtained by ADHD participants into percentiles, based on the Gaussian distribution curve. This transformation is done using specific correction tables for each test that refer to the standardization of the instrument in the normal Italian population (see cited references of normative values for details). This procedure includes correction factors such as age, gender and educational level. Following the guidelines of the Swiss Association of Neuropsychologists, percentiles have the advantage of characterizing a performance as pathological (percentile ≤5°), borderline (percentile >5 and ≤16°) or normal (percentile >16°), as a function of specific correction factors for the reference population. Accordingly, the results of each test considered was organized into the categories “Pathological,” “Borderline” and “Normal.” For the assessment of the differences based on socio-demographic characteristics, the categories “Pathological” and “Borderline” were merged and compared to the category “Normal.” This binary classification of the results obtained at psychometric tests has a crucial clinical value for the characterization of the disorders. In fact, a borderline performance in a patient who has no concomitant neurological or psychiatric disorders other than ADHD, is considered equally pathological and therefore symptomatic and specific of ADHD cognitive impairment. Expecting cell counts below 5, gender differences were assessed using the Fisher exact test. Instead, differences regarding age and years of education were assessed using the Mann–Whitney test because of the limited sample size. For the same reason, the statistical significance threshold was set at 10%. All statistical analyses were performed with Stata/IC 16.0 (StataCorp, 4,905 Lakeway Drive, College Station, Texas, United States).

## Results

Forty adult ADHD patients, aged between 18 and 55 years old, were recruited into the study (between February 2019 and May 2022). However, two patients did not give their consent to the re-use of health-related personal data; therefore, the total sample size is 38 participants (N = 38; 13 females and 25 males). The mean age is 28.3 ± 9.7 years old, while the mean number of education years is 11.6 ± 3.1.

For clinical reasons there are few missing data on neuropsychological tests (i.e., test not administered due to lack of time, patient too tired, choice of another complementary and/or equivalent test), while some BIS-11 self-questionnaires were not completed correctly by several patients (or were not completed at all). For this reason, the total sample for this scale is N = 27.

Table 1 reports the results of the tests according to the three categories of interest. Most of ADHD patients were classified as “Normal” in all *TAP Go/No-go* subscores, *ROCFT_copy*, *d2-R errors*, *5-points number of drawings* and *5-points errors*. Instead, patients were mostly classified as “Pathological” or “Borderline” in the *speed of execution* and *concentration performance* subscores of the *d2-R test*, in the *strategies of the 5-points test* and in the self-questionnaire *BIS-11* (mainly evidenced in the *attentive-cognitive_impulsivity* and *planning_impulsivity* subscales rather than in the *motor_impulsivity* domain).

**Table 1:** Results of the tests performed.

Test	Sample size (*n*)	Pathological (*n*)	Borderline (*n*)	Normal (*n*)

d2-R_RE (speed of execution)	37	17	7	13
d2-R_PC (concentration performance)	37	14	6	17
d2-R_errors	37	6	5	26
TAP_Go/Nogo_RT (reaction time)	38	5	6	27
TAP_ Go/Nogo_errors	38	5	4	29
TAP_ Go/Nogo_omissions	38	2	3	33

5-points_number_of_drawings	34	3	8	23
5-points _strategies	34	10	6	18
5-points _errors	34	1	0	33

ROCFT_copy	32	1	3	28

BIS11_total	27	12	12	3
BIS11_attentive-cognitive_impulsivity	27	20	5	2
BIS11_motor_impulsivity	27	10	3	14
BIS11_planning_impulsivity	27	16	4	7

The evaluation of the differences in the test’s results, according to socio-demographic characteristics (i.e., gender, age and years of education), did not produce any statistically significant results for gender. Moreover, it should be noted that a small sample size (N = 38) could be correlated to an increase of type II error in the statistical test (i.e., the fact of incorrectly assessing the lack of significance in the relation between a socio-demographic factor and a test score). On the other hand, contrary, we found 2 significant relationships for age and 3 for years of education. These statistically significant relationships are presented in Table 2, where the category-specific medians and interquartile ranges are reported together with the results of the Mann–Whitney test. Patients classified as “Pathological” or “Borderline” in the *TAP Go/No-go errors* and in the *5-points number of drawings* were significantly younger than the others (value of *p* = 0.009 and value of *p* = 0.042, respectively). Regarding education, “Pathological” and “Borderline” patients were characterized by a significantly lower number of years of education according to the results of the *d2-R speed of execution* (value of *p* = 0.071) and *d2-R errors* (value of *p* = 0.017) tests, while the opposite consideration yields for the self-administered questionnaire *BIS-11 planning impulsivity* (value of *p* = 0.006).

**Table 2:** Results of the bivariate analysis.

	Test result’s category		Mann–Whitney test result
	Pathological or bordeline	Normal	
*Age (years): Median (IQR)*			
TAP_ Go/Nogo_errors	20.0 (8.5)	27.0 (13.5)	*z* = −2.54^***^
5-points_number_of_drawings	23.0 (6.0)	28.0 (16.0)	*z* = −2.03^**^
*Education (years): Median (IQR)*			
d2-R_RE (speed of execution)	9.5 (4.0)	13.0 (3.5)	*z* = −1.81^*^
d2-R_errors	9.0 (3.0)	13.0 (4.5)	*z* = −2.35^**^
BIS11_planning_impulsivity	12.5 (3.8)	9.0 (1.0)	*z* = 2.69^***^

*** *p*-value < 0.01;

** *p*-value < 0.05;

* *p*-value < 0.10.

## Discussion

Accordance with the present literature, many of the adult ADHD patients recruited had attention and processing speed deficits (Onandia-Hinchado et al., 2021). The d2-R neuropsychological test might be considered a sensitive tool to identify the attentional dysfunction of this clinical population. Instead, concerning executive functioning, only some of them had a reduced performance in inhibitory control, planning, strategy and monitoring/updating. In this regard we observed that for the adult the copy of the ROCFT, is not a sensitive tool as the child one (Shin et al., 2003; Molitor et al., 2019). Since the ADHD patients recruited had a normal IQ, this could be related to the self-acquisition of drawing planning strategies learned through time and schooling (Canela et al., 2017; Kysow et al., 2017). Instead, the strategy subscore of the 5-points test revealed an inappropriate or reduced use of strategies in half of the patients. This confirms that this neuropsychological test is more adequate and sensitive for the adult ADHD population, revealing actual EFs disfunction. Furthermore, the BIS-11 self-administered questionnaire showed that most of the participants exhibited and perceived impulsive behaviors in everyday life. Accordingly, the most critical items were related to the attentive-cognitive and non-planning impulsivity (for example, “I do not pay attention” or “I have racing thoughts” or “I concentrate easily” or “I have outside thoughts when thinking” and “I plan tasks carefully” or “I say things without thinking” or “I get easily bored when solving thought problems”). The phenomena of attentional, cognitive and planning impulsivity were however found only in a few patients at TAP Go/No-go. All together, these findings are also consistent with the previous literature, supporting the highlighted lack of correlation between executive functioning and neuropsychological tests, often not adapted and not enough ecological for adult population (Barkley and Murphy, 2011).

Although many studies have shown a gender difference in the attentive-executive functioning of ADHD patients, highlighting predominantly attentional problems in females (Stibbe et al., 2020) and impulsivity and inhibitory control in males (Carucci et al., 2022), in this retrospective study we did not find any significant gender-related difference. This result could be linked to our limited sample size or explained by the fact that most of these evidences are based on children or adolescent (Carucci et al., 2022), while our study population is based on adults. Furthermore, it would also seem that females have a higher risk of having ADHD in the absence of “overwhelming” symptoms that lead to the request for help (Fraticelli et al., 2022). For this reason, perhaps the women who consulted our service, and who consequently were recruited into this study, may represent the group of females with a more pronounced symptomatology and therefore more similar to that of males, thus determining the founded similar socio-cognitive profile. Finally, consistently with Stibbe et al. (2020), we did not find gender differences even on self-assessment scale, such as the BIS-11 which assess the current impulsive symptomatology. Curiously, the analyses showed that patients with a higher level of education perceive and report a greater impairment relative to planning impulsiveness. This finding can have several explanations: the most comprehensive assumed that more cultured ADHD patients are also more aware of their functional limitations as they are confronted with educational and professional situations that require greater planning skills.

The bivariate analysis also evidenced that higher educated ADHD participants were more rapid and produced less errors in the d2-R test. Additionally, younger ADHD patients did more errors in the Go/No-go subtest of the TAP and produced less drawings on the 5-points test. Subsequently, this highlights that both the level of education and the chronological age influence the attention-executive performance of ADHD adults. It would therefore seem that younger ADHD adult patients have a higher level of impulsivity than older ones, which is reflected in a greater number of errors in the Go/No-go of TAP. Younger patients also manifested more difficulties in strategic planning and monitoring, which is reflected in the fewer number of drawings produced in the 5-point test. Contrariwise, a higher level of education allows adult ADHD patients to be faster and more performing, making even fewer distracting errors and/or omissions in a “scholastic” paper-pencil test such as the d2-R.

These new and interesting results are in line with the literature on the subject. In fact, it is well known that the impulsive component in ADHD is more overwhelming in children and adolescents and tends to decrease in adulthood (Willoughby, 2003; Langberg et al., 2008). In this sense, it would seem that in young adults there are still some aftermaths of this attitude which will tend to decrease more and more with advancing age. This is most likely related to compensatory strategies learned with advancing adulthood; theory also confirmed by a study with functional MRI (Dillo et al., 2010). The acquisition of experience is a crucial point especially for the development of specific strategies related to attention, planning and monitoring/updating. Indeed, exactly as theorized for the change in sensitivity of the ROCFT between childhood and young adulthood, ADHD adults do not stop acquiring and learning new compensatory strategies (Canela et al., 2017; Kysow et al., 2017), which can effectively help them to counterbalance the attention-executive difficulties typical of the disorder. Finally, as highlighted in our analysis, this process is potentially favored by the experience accumulated over the years and a higher level of education.

Limitations of the present study are the restricted sample size and the absence of a control group. Consequently, the interpretation of the results must be made with extreme caution, because some significant relations may be missing (due to the aforementioned type II errors) and also the sample could be not representative of the entire adult ADHD population. Therefore, the discussed findings must be considered as preliminary, to be corroborated with further evidence based on higher sample sizes, in order to ensure their correct generalization. Anyway, even considering the sample size, the highlighted significant relationships between cognitive performance and socio-demographic factor have a good statistical power. Furthermore, the transformation procedure employed to convert raw score into percentiles, based on specific correction tables for each test that refer to the standardization of the instrument in the normal Italian population, allows to compare the performance of our ADHD participants with a specific reference sample, as if this were a generic control group. A further limitation is the small number of tests administered to assess attention and EFs. In particular, it would have been appropriate to use additional tests to differentiate the functioning of reasoning and planning, defined as “cold” EF, from “hot” EF involving emotions (Chan et al., 2008). Then, other attention measures, such as the TAP divided attention or distractibility subtests could have been considered, together with complementary executive measures, such as the Wisconsin Card Sorting Test (WCST; Heaton, 1981) or the Iowa Gambling Task (IGT; Bechara, 2007), which evaluate “hot” EF as decision making process. Moreover, other self-report measures could have been employed, such as the Behavior Rating Inventory of Executive Functions (BRIEF-A; Roth et al., 2005), the Adult Executive Functioning Inventory (ADEXI; Holst and Thorell, 2018) or the Mind Wandering Excessively Scale (MEWS; Mowlem et al., 2019). However, we had to rely on the psychometric measures available and administered clinically according to the clinical procedure for the diagnosis of ADHD in adults developed in our service. In this sense, the main strength of our study is the selection of patients, enrolled according to an accurate diagnostic process and free from pharmacotherapy that could have affected their cognitive performance.

## Conclusion

This retrospective case series study confirms that in order to correctly test and evaluate the various aspects of EFs, it is necessary to use appropriate neuropsychological tests. In this sense, our results show that the use of ROCFT is not adequate in an adult ADHD population as it is for childhood ADHD. Our findings therefore highlight that to evaluate the planning, strategy and monitoring/updating skills in adult ADHD patients it is necessary to use more suitable neuropsychological tests such as the 5-points test. In addition, our result suggest that it is clinically important to always employ self-administered questionnaires to better evaluate the functional impact on everyday life of inattentive and dysexecutive symptoms. Furthermore, our choice to directly investigate how the cognitive performance of adults with ADHD varies according to specific socio-demographic factors, has allowed us to highlight the possibility that adult ADHD is not a stable disorder, but is constantly changing. Indeed, related attention-executive deficits may be influenced and partially compensated by experience (i.e., advancing age) and a higher level of education. This could underlie the development of specific compensatory strategies that allow ADHD adults to counterbalance, at least in part, the difficulties related to their clinical condition. This aspect must therefore be taken into consideration during the neuropsychological assessment of adult ADHD patients, especially for those who are older and with a higher level of education. In this sense, it seems of fundamental importance to also use self-administered questionnaires, which can help highlight attentional and executive difficulties that may not be so overwhelming on neuropsychological tests, because they are globally compensated by specific psycho-behavioral and cognitive strategies.

From this perspective, it would be particularly interesting to re-evaluate ADHD adults over the years, observing possible changes in the cognitive performance. Further investigations involving a specific control group and a larger cohort of non-medicated patients, to avoid possible effects related to the use of pharmacotherapy over time, could accordingly lead to more powerful and generalizable results. Moreover, it would be relevant to link neuropsychological performances of ADHD adults with specific data of functional brain imagery, to verify if the cognitive changes are linked to neural modulations or if they are simply the result of behavioral and cognitive strategies implemented by patients over the years. This would bring new evidence to complement the review of Fassbender and Schweitzer (2006).

Finally, following the meta-analysis of Bora and Pantelis (2016), it would be interesting to include specific measures for social cognition, evaluating whether the observed attentive-executive dysfunction may also have an impact on social skills and how this varies according to socio-demographic factors. Indeed, the preponderant role of attention and EFs in social cognition dysfunction in adults with ADHD has already been demonstrated (Mary et al., 2016; Tatar and Cansiz, 2022), but additional evidence is needed on how these aspects vary over time and if there are other variables that modulate social functioning. Also for the assessment of social cognition, as for the attentive-executive examination, we esteem it is of fundamental importance to couple neuropsychological tests with self-administered questionnaires, such as the Difficulties in Emotion Regulation Scale (DERS; Gratz and Roemer, 2004) or the Interpersonal Reactivity Index (IRI; Davis, 1980).

## Data availability statement

The original contributions presented in the study are included in the article/supplementary material, further inquiries can be directed to the corresponding author.

## Ethics statement

The studies involving human participants were reviewed and approved by Cantonal Ethics Committee, Repubblica e Cantone Ticino (project-ID 2022-01085 CE 4122). The patients/participants provided their written informed consent to participate in this study.

## Author contributions

MC conceptualized, designed and supervised the whole study, revised and corrected the raw data of the participants and drafted the manuscript. GZ, EB, and SR evaluated and screened patients and also collected data. ES performed the statistical analysis and wrote part of the method and the results. AI reviewed the final version of the manuscript and collaborated to the submission. LM designed the study, reviewed the final version and submitted the manuscript. LS conceptualized, designed and supervised the whole study, wrote part of the manuscript and reviewed the final version. All authors read and accepted the final version of the paper and reported above for publication was completed and contributed to the article and approved the submitted version.

## Funding

Open access funding was provided by the Università Della Svizzera Italiana.

## Conflict of interest

The authors declare that the research was conducted in the absence of any commercial or financial relationships that could be construed as a potential conflict of interest.

## Publisher’s note

All claims expressed in this article are solely those of the authors and do not necessarily represent those of their affiliated organizations, or those of the publisher, the editors and the reviewers. Any product that may be evaluated in this article, or claim that may be made by its manufacturer, is not guaranteed or endorsed by the publisher.
