# Erratum: Attentive-executive functioning and compensatory strategies in adult ADHD: A retrospective case series study

**DOI:** 10.3389/fpsyg.2023.1176498

**Published:** 2023-03-09

**Authors:** Frontiers Production Office

**Affiliations:** Frontiers Media SA, Lausanne, Switzerland

**Keywords:** adult ADHD, cognition, executive functions, attention, socio-demographic factors

An omission to the funding section of the original article was made in error. The following sentence has been added: “Open access funding was provided by the Università Della Svizzera Italiana.”

The original article has been updated.

